# Excessive Accumulation of Ca^2 +^ in Mitochondria of Y522S-RYR1 Knock-in Mice: A Link Between Leak From the Sarcoplasmic Reticulum and Altered Redox State

**DOI:** 10.3389/fphys.2019.01142

**Published:** 2019-09-13

**Authors:** Marta Canato, Paola Capitanio, Lina Cancellara, Luigi Leanza, Anna Raffaello, Denis Vecellio Reane, Lorenzo Marcucci, Antonio Michelucci, Feliciano Protasi, Carlo Reggiani

**Affiliations:** ^1^Department of Biomedical Sciences, School of Medicine and Surgery, University of Padova, Padua, Italy; ^2^Department of Biology, University of Padova, Padua, Italy; ^3^Center for Advanced Studies and Technology, Università degli Studi “G. d’Annunzio” Chieti–Pescara, Chieti, Italy; ^4^Department of Medicine and Aging Sciences, Università degli Studi “G. d’Annunzio” Chieti–Pescara, Chieti, Italy; ^5^Institute for Kinesiology Research, Science and Research Center of Koper, Koper, Slovenia

**Keywords:** excitation–contraction coupling, mitochondria, reactive oxygen species, ryanodine receptor, malignant hyperthermia

## Abstract

Mice (Y522S or YS), carrying a mutation of the sarcoplasmic reticulum (SR) Ca^2+^ release channel of skeletal muscle fibers (ryanodine receptor type-1, RyR1) which causes Ca^2+^ leak, are a widely accepted and intensively studied model for human malignant hyperthermia (MH) susceptibility. Since the involvement of reactive oxygen species (ROS) and of mitochondria in MH crisis has been previously debated, here we sought to determine Ca^2+^ uptake in mitochondria and its possible link with ROS production in single fibers isolated from flexor digitorum brevis (FDB) of YS mice. We found that Ca^2+^ concentration in the mitochondrial matrix, as detected with the ratiometric FRET-based 4mtD3cpv probe, was higher in YS than in wild-type (WT) fibers at rest and after Ca^2+^ release from SR during repetitive electrical stimulation or caffeine administration. Also mitochondrial ROS production associated with contractile activity (detected with Mitosox probe) was much higher in YS fibers than in WT. Importantly, the inhibition of mitochondrial Ca^2+^ uptake achieved by silencing MCU reduced ROS accumulation in the matrix and Ca^2+^ release from SR. Finally, inhibition of mitochondrial ROS accumulation using Mitotempo reduced SR Ca^2+^ release in YS fibers exposed to caffeine. The present results support the view that mitochondria take up larger amounts of Ca^2+^ in YS than in WT fibers and that mitochondrial ROS production substantially contributes to the increased caffeine-sensitivity and to the enhanced Ca^2+^ release from SR in YS fibers.

## Introduction

Malignant hyperthermia is a rare life-threatening response triggered by exposure to halogenated/volatile anesthetics commonly used during surgery interventions (halothane, isofluorane, etc.). MH crises are primarily caused by a sustained and uncontrolled release of Ca^2+^ from the SR of skeletal muscle fibers (MacLennan and Phillips, 1992). A large percentage of MH cases have been associated to mutations in the *RYR1* gene, encoding for a large protein of about 565 kDa that forms the tetrameric structure of the SR Ca^2+^ release channel of skeletal muscle, i.e., the RyR1 (Galli et al., 2006). RyR1 plays a central role in EC coupling, the mechanism that allows transduction of the action potential into Ca^2+^ release from the SR in muscle (Schneider, 1994; Franzini-Armstrong and Protasi, 1997).

The murine strain carrying the mutation Y524S in RyR1 (YS knock-in mice), corresponding to the human Y522S mutation, has been widely studied in the last 10 years as a model for MH susceptibility as YS mice suffer lethal episodes when exposed to halothane, heat, and also physical exertion (Chelu et al., 2006; Durham et al., 2008; Michelucci et al., 2017a). Since the first studies published by Hamilton and coworkers it was clear that the mutation causes an increased sensitivity of the RyR1 channel to caffeine, isoflurane, and temperature (Chelu et al., 2006; Durham et al., 2008). The Y522S mutation determines a shift of the voltage dependence of activation and inactivation of Ca^2+^ release to more negative voltage compared to WT channel (Andronache et al., 2009; Zullo et al., 2018) and impairs the Ca^2+^-dependent inactivation of the release (Manno et al., 2013). The opening probability of RyR1 can be further increased by *S*-nitrosylation of several cysteines of RyR1 (Aracena-Parks et al., 2006; Sun et al., 2008, 2013). Indeed, as shown by Hamilton and coworkers (Durham et al., 2008), RyR1 cysteine nitrosylation can occur under conditions such as exposure to anesthetics and environmental heat. RyR1 nitrosylation has been proposed to create a feed forward loop in YS muscles (Durham et al., 2008), where SR Ca^2+^ leakage from the mutated RyR1 channel results in increased cytosolic Ca^2+^, that in turn produces nitrosative modifications of the RyR1 channel via generation of reactive species of oxygen and nitrogen (ROS and RNS). The nitrosative modifications further enhance channel permeability and Ca^2+^ leakage. Such a feed-forward loop has been considered the key mechanism for development of the MH crises (Durham et al., 2008). In accordance with this view, the YS mice have become a model to study interventions to control the crises, based on antioxidant compounds as *N*-acetylcysteine or NAC (Durham et al., 2008) or specific molecules controlling Ca^2+^ release as AICAR (Lanner et al., 2012) and possibly also Ca^2+^ entry such as dantrolene or azumulene (Zhao et al., 2006; Michelucci et al., 2017a).

Also mitochondria are affected by the RyR1 Y522S mutation. Indeed, their oxidative damage, e.g., lipid peroxidation, and progressive degeneration have been reported as the mechanism leading to fiber damage and formation of cores in muscle fibers of YS mice. Swollen mitochondria, with ultrastructural damage of cristae, are present in 2 months old YS mice (Durham et al., 2008) and become more abundant with increasing age (Boncompagni et al., 2009a). As mitochondria might participate in ROS/RNS generation, their contribution to RyR1 nitrosylation, Ca^2+^ release, and hypermetabolism during hyperthermic MH crises has been hypothesized (Durham et al., 2008). However, the relevance of the mitochondrial contribution to the free radicals overproduction during MH crises is still debated (Lanner et al., 2012), because several other possible sources of ROS/RNS are available in muscle fibers as NOX, NOS, PLA2, and xanthine oxidase (Powers and Jackson, 2008; Lanner et al., 2012). Specifically, it is not known yet if Ca^2+^ uptake and ROS generation in the mitochondria of YS muscle fibers is abnormal during regular contractile activity and/or during hyperthermic crisis.

In the present study, we adopted a widely accepted experimental model, i.e., single fibers enzymatically dissociated from FDB muscles, and investigated whether mitochondrial Ca^2+^ uptake is altered in YS muscle. In addition, we tested the hypothesis that entry of Ca^2+^ into mitochondria may influence mitochondrial ROS generation and the global oxidative/nitrosative stress of YS fibers. The results obtained in this study show an increased Ca^2+^ entry in the mitochondria of YS fibers and support the view that Ca^2+^ accumulation in the mitochondrial matrix and mitochondrial ROS production are relevant to the feed forward mechanism which enhances sensitivity to caffeine and Ca^2+^ release through the mutated RyR1 channel.

## Materials and Methods

### Animals

Experiments were carried out in heterozygous RYR1^Y524S/WT^ mice (hereafter indicated simply as YS; *n* = 12), and in WT mice C57BL/6J (hereafter indicated as WT; *n* = 12) of 6–8 weeks of age. YS mice were generated as previously described (Chelu et al., 2006) and generously provided by Dr. S. L. Hamilton (Baylor College of Medicine, Houston, TX, United States). Mice were housed in cages at 22°C in a 12-h light/dark cycle and had free access to water and food. All experiments were conducted according to the Directive of the European Union 2010/63/UE. All animal protocols were approved by the Committee on the Ethics of Animal Experiments of the University of Chieti (Permit Number: 40). For each group (WT and YS), four mice were used for the Fura-2 experiments, three for mitochondrial cameleon transfection, three for SR cameleon transfection, and two for MCU silencing experiments. Fragments of muscles were used for Western blot (WB) and PCR experiments.

### MCU Silencing Experiments

Silencing of MCU was achieved by transfection of plasmids coding for shRNA targeting the MCU mRNA and for a fluorescent marker, either ZsGreen or mCherry (pZac-U6-shMCU-ZsGreen or pZac-U6-shMCU-mCherry), as previously done (Mammucari et al., 2015). The control muscles were electroporated with plasmids encoding shRNA against the Luciferase mRNA and a fluorescent marker (pZac-U6-shLuc-ZsGreen or pZac-U6-shLuc-mCherry).

pZac-U6-shLuc-ZsGreen was purchased from the University of Pennsylvania Vector Core (Philadelphia, PA, United States). For pZac-U6-shMCU-ZsGreen, the MCU shRNA sequence was cloned into *Bam*HI–*Eco*RI sites of pZac-U6-shLuc-ZsGreen with the primers 5′-GATCGGATCCGAGATGACCGTGAATCTTCAAGAGAGATT CACGGTCATCTCGGATCTTTTTG-3′ (forward) and 5′-AA TTCAAAAAGATCCGAGATGACCGTGAATCTCTCTTGAA GATTCACGGTCATCTCGGATCC-3′ (reverse).

For pZac-U6-shMCU-mCherry and pZac-U6-shluc-mCherry, ZsGreen cassettes of pZac-U6-shMCU-ZsGreen and of pZac-U6-shluc-ZsGreen were substituted with the mCherry cassette of pmCherry-N1 (Clontech Laboratories, Mountain View, CA, United States) at *Nhe*I–*Not*I sites.

Plasmids were transfected in FDB muscles by electroporation as described previously (Canato et al., 2010; Scorzeto et al., 2013). Briefly, WT and YS mice were anesthetized by intraperitoneal injection of 100 mg/kg ketamine, 10 mg/kg xylazine, and 3 mg/kg acepromazine. Hind limb footpads of anesthetized mice were injected subcutaneously with bovine hyaluronidase (7 μl/foot, 2 μg/μl), and 1 h later, with 15 μg of plasmidic DNA using a 30-gauge needle. The footpad was then electroporated using subcutaneous electrodes. FDB muscle fibers were isolated and plated 8–12 days after electroporation.

### Isolation of Single Fibers From Flexor Digitorum Brevis Muscle

Single fibers were prepared from FDB muscles of YS and WT mice according to a modified collagenase-dissociation method previously described (Defranchi et al., 2005). Briefly, FDB muscles were dissected and incubated at 37°C for 1–2 h in 0.2% collagenase in Tyrode’s solution (Sigma–Aldrich, Milano, Italy) containing 10% fetal bovine serum (Sigma–Aldrich, Milano, Italy). After digestion, collagenase was removed by 3 min wash with Tyrode’s solution, then muscles were incubated for 15 min in Tyrode’s solution containing 10% fetal bovine serum to completely block the collagenase activity, and finally washed again for 3 min in Tyrode’s solution. Fibers were dissociated using Pasteur pipettes of decreasing diameter, plated on laminin-coated glass coverslips positioned at the center of 35-mm dishes, and incubated overnight at 37°C in Tyrode’s solution supplemented with 10% heat-inactivated FBS and 1% antibiotic–antimycotic solution (10,000 units penicillin, 10 mg streptomycin, and 25 μg amphotericin B) (Sigma–Aldrich, Milano, Italy).

### Electrical Stimulation and Caffeine Administration in Single FDB Fibers

Plated single FDB fibers were subjected to electrical field stimulation via platinum electrodes. Electrical pulses were delivered a 0.5 Hz for 5 min, then trains of 2 s duration and 60 Hz frequency were delivered. Caffeine was added either in a single shot to reach the concentration in the medium of 10 or 20 mM or in subsequent shots to reach increasing concentrations (2, 4, 6, and 10 mM) according to the cumulative dose procedure. This last procedure made it possible to reproduce on single muscle fibers a dose–response curve, reminiscent of the *in vitro* contracture test (IVCT) used for diagnostic purpose (Melton et al., 1989). In a specific series of experiments, muscle fibers were incubated in the presence of the mitochondria-targeted antioxidant, specific scavenger of mitochondrial superoxide, 2-(2,2,6,6-tetramethylpiperidin-1-oxyl-4-ylamino)-2-oxoethyl (MitoTEMPO) triphenyl phosphonium chloride (Sigma–Aldrich, Milano, Italy) which was added at the concentration 50 μM either 24 or 1 h before the experiment (see for reference Davuluri et al., 2016; Bae et al., 2018).

### Recording of Fura-2 Signals

Twenty-four hours after dissociation, single FDB fibers were incubated for 30 min at 37°C with 5 μM Fura-2 acetoxymethyl ester (Fura-2 AM; Invitrogen, Monza, Italy) in a solution of the following composition, 125 mM NaCl, 5 mM KCl, 1 mM MgSO4, 1 mM KH2PO4, 5.5 mM glucose, 1 mM CaCl2, 20 mM HEPES, containing 1% bovine serum albumin (BSA), pH 7.4 (incubation buffer). After Fura-2 AM loading, the FDB fibers were washed twice for 10 min in the incubation buffer without Fura-2 AM and BSA at 37°C to retain the indicator in the cytosol, and then immersed in imaging buffer (125 mM NaCl, 5 mM KCl, 1 mM MgSO_4_, 1 mM KH_2_PO_4_, 5.5 mM glucose, 1 mM CaCl_2_, 20 mM HEPES with 50 μM *N*-benzyl-*p*-toluene sulfonamide or BTS). All reagents were purchased at Sigma–Aldrich (Milano, Italy), but BTS purchased at ThermoFisher Sci (Monza, Italy). After a minimum of 30 min, calcium signals were recorded using a dual-beam excitation fluorescence photometry setup (IonOptix Corp., Westwood, MA, United States) at 25°C. Measurements were expressed as the ratio of the emission at 510 nm with excitation at 360 and 380 nm. Recordings were analyzed with the IonWizard software (IonOptix Corp., Westwood, MA, United States). For silencing experiments only fibers effectively transfected and thus recognized by their red fluorescence due to mCherry expression (see above) were recorded.

**FIGURE 1:**
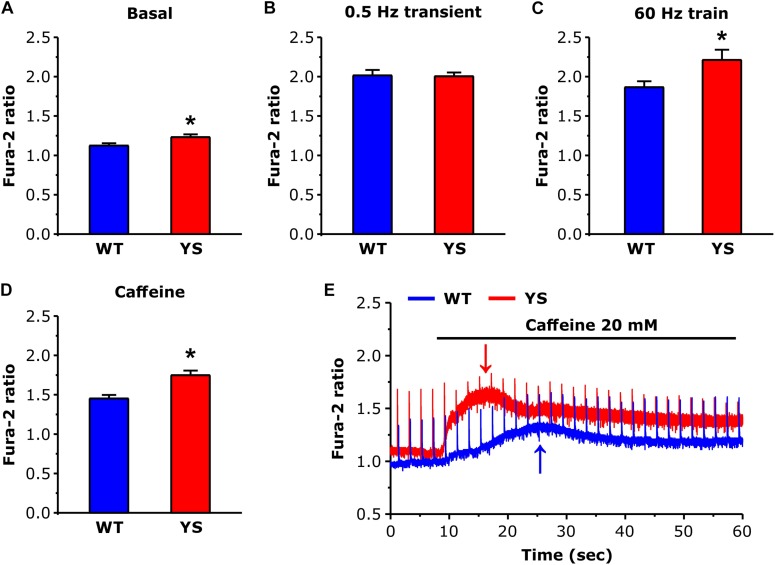
Cytosolic free Ca^2+^ concentrations at rest and during contraction as determined by Fura-2. **(A)** Basal resting Ca^2+^ levels during low frequency stimulation at 0.5 Hz. **(B)** Ca^2+^ levels at the peak of transients elicited by electrical stimulation at 0.5 Hz (average of 10 transients in each fiber). **(C)** Values of Ca^2+^ levels reached at the end of a train of electrical stimulation at 60 Hz, 2 s duration (average of the last 100 ms). **(D)** Values of basal Ca^2+^ levels reached at the first peak after exposure to 20 mM caffeine. **(E)** Representative recordings of Fura-2 fluorescence ratio in WT and YS fibers during caffeine administration, note that the first peak of basal Ca^2+^ concentration (pointed by arrows) occurs earlier and is higher in YS compared to WT muscle fibers and that in YS fibers the basal Ca^2+^ concentration remains high. Data are shown as means ± SEM; ^∗^*p* < 0.05. Panels **A–C**: *n* = 33 WT and 33 YS fibers; panel **D**: *n* = 13 WT and 13 YS fibers.

The basal Fura-2 ratio level was measured in the interval between transients elicited by electrical stimulations at 0.5 Hz, the amplitude of the transients elicited by electrical stimulation was measured at their peaks in 0.5 Hz twitches and as average of the last 100 ms of stimulation in 60 Hz 2 s unfused tetani. The amplitude of the response to caffeine was measured at the first peak of basal (between transients) calcium concentration after compound addition (see arrows in Figure 1E).

### Detection of Free Ca^2+^ in SR and Mitochondrial Matrix

Measurements of free Ca^2+^ concentrations in mitochondrial matrix and SR lumen were performed using cameleon Ca^2+^ probes 4mtD3cpv (Palmer et al., 2006) and, respectively, D1-ER (Palmer et al., 2004), kindly donated by Dr. R. Y. Tsien (University of California, San Diego, CA, United States). The probes were expressed in FDB muscles by electroporation of plasmid vectors as described above (see also Canato et al., 2010; Scorzeto et al., 2013).

Mitochondrial and SR free Ca^2+^ levels were determined from the YFP/CFP ratio of the cameleon probes using an inverted fluorescence microscope (Eclipse-Ti; Nikon Instruments, Firenze, Italy) equipped with the perfect focus system (PFS). Fibers were placed in a chamber containing imaging buffer, mounted on the movable stage of the microscope and temperature was set at 25°C. Excitation of the fluorophore was performed by means of a Hg arc lamp using a 435-nm filter (10-nm bandwidth). YFP and CFP intensities were recorded by means of a cooled CCD camera (C9100-13; Hamamatsu-Photonics Italia, Roma, Italy) equipped with a 515-nm dichroic mirror at 535 (40-nm bandwidth) and 480 nm (30-nm bandwidth), respectively. Two images of 256 × 128 pixels each, corresponding, respectively, to YFP and CFP light emissions, were collected with a time resolution of 9 ms. YFP and CFP intensities were corrected for background and the ratio *R* was defined as follows: *R* = (YFPfiber − YFPbackground)/(CFPfiber − CFPbackground). For silencing experiments only fibers effectively transfected and recognized by their red fluorescence due to mCherry expression (see above) were recorded. The basal ratio level was measured just at the beginning of the recordings, the amplitude of the transients elicited by electrical stimulation was measured at their peaks, and the amplitude of the response to caffeine stimulation was measured at the first peak of calcium concentration (see arrows in Figure 1E).

### Detection of Reactive Oxygen Species With MitoSOX

Twenty-four hours after dissociation, FDB fibers were incubated for 20 min in 2 ml Dulbecco’s Phosphate-Buffered Saline (D-PBS) containing 1 μM MitoSOX^TM^ Red (Invitrogen, Monza, Italy) at 37°C in a tissue culture incubator. Fibers were then washed twice with D-PBS and two further times with Tyrode’s solution. Fibers were maintained in 2 ml Tyrode’s solution at 25°C during the experimental protocol. Caffeine 20 mM was added and fluorescence recorded for 15 min. Images before and after caffeine addition were obtained with Leica DMI6000 confocal microscopy. MitoSOX^TM^ Red excitation was performed at 488 nm, and emission was collected at 580 nm. Data were analyzed by Image J software (see Leanza et al., 2017) and values at each time were expressed as percent of the value before caffeine administration. For silencing experiments only fibers effectively transfected and recognized by their green fluorescence due to zsGreen expression (see above) were recorded.

### Western Blot and Quantitative Real-Time PCR

Expression of proteins of the mitochondrial membrane and proteins involved in intracellular calcium dynamics were studied by WB and by RT quantitative PCR. For this analysis, not only FDB but also Soleus (SOL) and extensor digitorum longus (EDL) were utilized.

Skeletal muscle samples (FDB, SOL, and EDL muscle) were collected from three age-matched WT and YS mice. Total RNA was extracted through mechanical tissue homogenization (Tissuelyser II, Qiagen, Milano, Italy) in TRIZOL reagent (Thermo Fisher Scientific, Monza, Italy), following manufacturer instructions. The RNA was quantified with Nanodrop (Thermo Fisher Scientific, Monza, Italy) and retro-transcribed with the cDNA synthesis kit SuperScript II (Thermo Fisher Scientific, Monza, Italy). Oligo(dT)12–18 primers (Thermo Fisher Scientific, Monza, Italy) were used as primers for first-strand cDNA synthesis with reverse transcriptase. The obtained cDNA was analyzed by Real-Time PCR using the IQ5 thermocycler (Biorad, Segrate, Italy) and the SYBR green chemistry (IQ Sybr Green Super Mix, Biorad, Segrate, Italy). All data were normalized to GAPDH expression and plotted in fold changes compared to WT mice as mean ± SEM. The oligonucleotide primers used are:

GAPDH:

Fw 5′-CACCATCTTCCAGGAGCGAG-3′
Rv 5′-CCTTCTCCATGGTGGTGAAGAC-3′

MCU:

Fw 5′-AAAGGAGCCAAAAAGTCACG-3′
Rv 5′-AACGGCGTGAGTTACAAACA-3′

MICU1:

Fw 5′-GTCGAACTCTCGGACCATGT-3′
Rv 5′-GTGCTAAGGTGCAGGAGGTG-3′

MICU2:

Fw 5′-TGGAGCACGACGGAGAGTAT-3′
Rv 5′-GCCAGCTTCTTGACCAGTGT-3′

MCUb:

Fw 5′-AGTTACCTTCTTCCTGTCGTTTGCG-3′
Rv 5′-CAGGGATTCTGTAGCCTCAGCAAGG-3′

Muscle samples were processed for WB as previously described (Mammucari et al., 2015; Vecellio Reane et al., 2016). Briefly, frozen muscles were mechanically lysed in Tissuelyser II (Qiagen, Milano, Italy) in the muscle lysis buffer (50 mM Tris pH 7.5, 150 mM NaCl, 5 mM MgCl_2_, 1 mM DTT, 10% glycerol, 2% SDS, 1% Triton X-100, Roche Complete Protease Inhibitor Cocktail, 1 mM PMSF, 1 mM NaVO_3_, 5 mM NaF, and 3 mM β-glycerophosphate). The lysate protein concentrations were determined spectrophotometrically by using Pierce *BCA* Protein Assay Kit (Thermo Fisher Scientific, Monza, Italy). For WB analyses, a total amount of 40 μg of protein were loaded and separated on a 4–12% linear gradient acrylamide gels (Thermo Fisher Scientific, Monza, Italy), then transferred onto nitrocellulose membranes using a semidry transfer protocol (BioRad, Segrate, Italy). Blots were blocked 1 h at room temperature with 5% non-fat dry milk (BioRad, Segrate, Italy) in TBS-tween (50 mM Tris, 150 mM NaCl, 0.01% Tween) solution and incubated over-night at 4°C with primary antibodies diluted in the blocking solution: anti-actin (SPM161, Santa Cruz Biotechnology, Dallas, TX, United States); anti-TOM20 (FL-145, Santa Cruz Biotechnology, Dallas, TX, United States); anti-MCU (AMAB91189, Sigma–Aldrich, Milano, Italy). Secondary antibodies (isotype-specific HRP-conjugated antibodies, BioRad, Segrate, Italy) were incubated 1 h at room temperature. Washes after antibody incubations were done on an orbital shaker, three times for 10 min each, with TBS-tween. Immunodetection was performed with the Chemiluminescence SuperSignal^TM^ West Pico Chemiluminescent Substrate kit (Thermo Fisher Scientific, Monza, Italy) and the signal acquisition was performed through the detection system Uvitec Mini HD9.

Quantification was based on BAP analysis, i.e., measurement of the Brightness Area Product (product of the area of the band by the average brightness subtracted local background after black-white inversion) after scanning the gels with the accuracy of 600 DPI. Actin was used as reference and the values of BAP for TOM20 were expressed as fraction of the BAP value of actin. Each determination was done in triplicate. In turn, TOM20 was used as a reference for MCU.

### Statistical Analysis

Data were expressed as means ± standard error of the mean (SEM). Comparison between YS and WT means were carried out by Student’s *t*-test, setting the statistical significance at ^∗^*p* < 0.05 and ^∗∗^*p* < 0.01. For data in Figure 5 comparisons were carried out by one-way ANOVA, followed by Tukey’s test. For data in Figure 7, multiple *t*-test was used and statistical significance was determined using the Holm–Sidak method, with α = 0.05. All statistical analysis and curve fitting were done with the software Graphpad Prism 7.

## Results

### Cytosolic Ca^2+^: Higher Levels in YS Compared to WT

We determined resting cytosolic free Ca^2+^ concentration with Fura-2 while FDB muscle fibers were continuously stimulated at low frequency (0.5 Hz). Basal resting Ca^2+^ levels were slightly, but significantly, higher in YS compared to WT fibers (Figure 1A). In contrast, no significant difference was present when we compared the average peak values of the transients (mean of 10 peaks in each fiber) induced by single electrical pulses at 0.5 Hz (Figure 1B). However, higher values of Fura-2 ratio were reached in YS compared to WT when fibers were stimulated with 2 s trains of electrical pulses at 60 Hz (Figure 1C). Finally, the first peak of cytosolic Ca^2+^ determined by exposure to 20 mM caffeine was significantly higher and earlier in YS compared to WT (Figures 1D,E).

### Free Ca^2+^ Concentrations in the SR Lumen: Greater Depletion in YS Compared to WT

**FIGURE 2:**
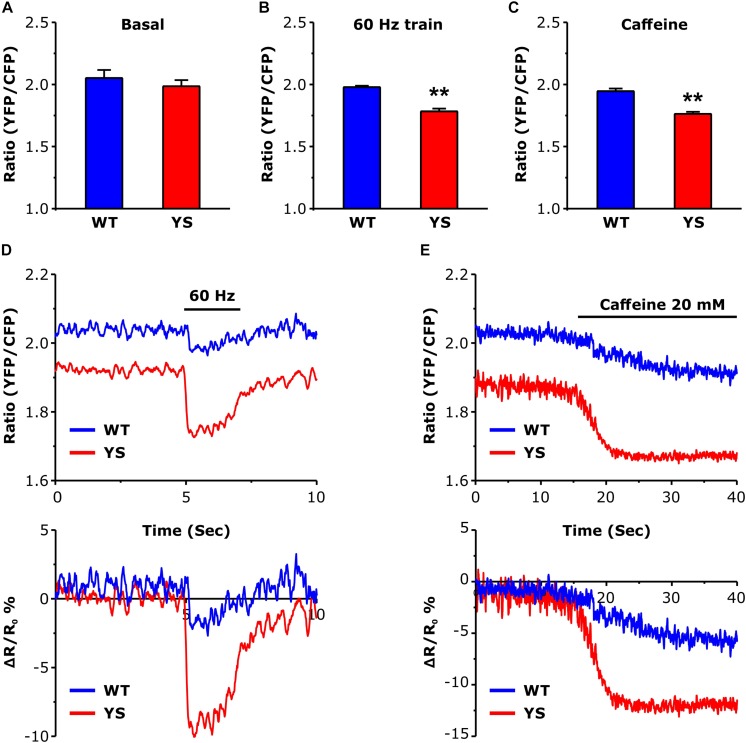
Intraluminal free Ca^2+^ concentration in the SR as determined by a YFP/CFP ratio of ER-D1 cameleon. **(A)** Basal resting Ca^2+^ levels in the SR lumen during low frequency stimulation at 0.5 Hz. **(B)** Intraluminal Ca^2+^ concentrations reached at the end of a train of electrical stimulation at 60 Hz, 2 s duration. **(C)** Intraluminal Ca^2+^ concentrations following exposure to 20 mM caffeine. **(D)** Representative traces of ER-D1 cameleon signal before, during, and after a train of electrical stimulation (indicated by horizontal line) of the 2 s pulse at 60 Hz. **(E)** Representative recordings of initial variations of ER-D1 cameleon signal before and after the administration of 20 mM caffeine (horizontal line indicates the time of the caffeine administration). Data are shown as means ± SEM; ^∗∗^*p* < 0.01. Panels **A** and **B**: *n* = 20 WT and 30 YS fibers; panel **C**: *n* = 7 WT and 7 YS fibers.

The higher levels reached by cytosolic free Ca^2+^ during electrical stimulation or caffeine administration (Figure 1) could in principle be accompanied by a greater depletion of the SR stores. The variations of intraluminal free Ca^2+^ concentration in the SR (Figure 2) were determined using the cameleon D1-ER, a FRET-based Ca^2+^ sensor specifically targeted to the SR lumen (Canato et al., 2010). While the resting basal levels were not significantly lower in YS compared to WT fibers (Figure 2A), a significant difference was detected during the Ca^2+^ release induced by 2 s trains of electrical stimulation at 60 Hz frequency (Figures 2B,D) or during exposure to 20 mM caffeine (Figures 2C,E), with SR depletion being significantly greater in YS than in WT fibers.

### Free Ca^2+^ Concentrations in the Mitochondrial Matrix: Higher Basal Level and Greater Increase in YS Compared to WT

**FIGURE 3:**
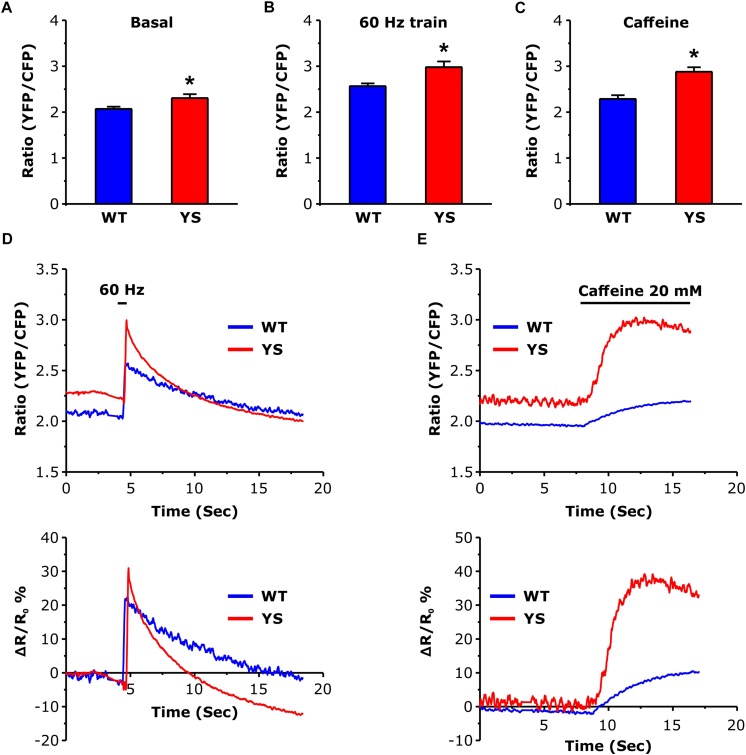
Free Ca^2+^ concentration in the mitochondrial matrix as determined by YFP/CFP ratio of 4mtD3cpv cameleon. **(A)** Basal resting Ca^2+^ levels in the mitochondrial matrix. **(B)** Peak of Ca^2+^ level in the mitochondrial matrix reached at the end of a train of electrical stimulation at 60 Hz, 2 s duration. **(C)** Peak of Ca^2+^ level in the mitochondrial matrix following exposure to 20 mM caffeine. **(D)** Representative traces of variations of YFP/CFP ratio of 4mtD3cpv cameleon signal before, during, and after a 2 s electrical stimulation at 60 Hz (horizontal line). **(E)** Representative recordings of initial variations of 4mtD3cpv cameleon signal before and after the administration of 20 mM caffeine (horizontal line indicates the time of the caffeine administration). Data are shown as means ± SEM; ^∗^*p* < 0.05. Panels **A** and **B**: *n* = 20 WT and 30 YS fibers; panel **C**: *n* = 7 WT and 7 YS fibers.

Measurement of the free Ca^2+^ concentration in the mitochondrial matrix represented one of the major aims of the present study. We determined free Ca^2+^ concentration in the mitochondrial matrix using the 4mtD3cpv cameleon, a FRET-based Ca^2+^ sensor specifically designed to be targeted to the mitochondrial matrix (Scorzeto et al., 2013). While no significant differences were detected in the basal luminal SR Ca^2+^ level (Figure 2), the basal resting Ca^2+^ level in the mitochondrial matrix was significantly higher in YS compared to WT fibers (Figure 3A). In addition, the peak values reached both during electrical stimulation (2 s pulses at 60 Hz) and during Ca^2+^ release induced by 20 mM caffeine administration were significantly higher in YS compared to WT (Figures 3B–E).

### Mitochondrial Volume: Increased in YS Compared to WT, With No Change in Mitochondrial Inner Membrane Ca^2+^ Transport

**FIGURE 4:**
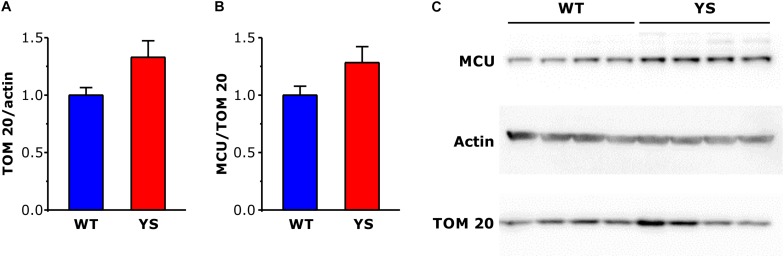
Expression levels of mitochondrial proteins TOM20 and MCU in FDB muscles determined by Western blot. **(A,B)** Bar plots of brightness area product (BAP) of the immunostained bands, showing expression levels of TOM 20 **(A)** in relation to α-actin taken as a loading control and MCU **(B)** in relation to TOM 20, taken as reference of mitochondrial volume. **(C)** Representative western blot gels of α-actin, TOM 20, and MCU. Data are shown as means ± SEM. Panels **A** and **B**: *n* = 8 in each column (four mice).

The higher values of Ca^2+^ concentration in the mitochondrial matrix could be a direct consequence of the greater Ca^2+^ release from SR, but could be in principle also due to changes in mitochondrial membrane permeability. We selected the outer membrane protein TOM20 and the myofibrillar protein alpha-actin as markers of mitochondrial and, respectively, myofibrillar content. Semiquantitative WB analysis showed a trend to increase in TOM20/actin ratio (*P* = 0.05), which suggests an increase in mitochondrial number or mitochondrial dimensions in YS compared to WT fibers of FDB muscles (Figure 4A). A similar increment was observed also in EDL and SOL, considered representative fast and slow muscles, respectively (data not shown).

We then analyzed some components of the mitochondrial Ca^2+^ transport system, among them the mitochondrial Ca^2+^ uniporter or MCU, its gatekeepers MICU1 and MICU2 and the dominant-negative isoform MCUb. The expression of MCU was determined by WB not only on FDB, but also on EDL and Soleus (data not shown) using TOM20 as a reference for the mitochondrial volume. The results showed no significant variations in the MCU/TOM20 ratio (Figure 4B), thus indicating that MCU abundance changed in parallel with the increase of mitochondrial volume.

We then determined by RT quantitative PCR the expression level of the MCU, its gatekeepers MICU1 and MICU2, and the MCU dominant-negative isoform MCUb. No significant difference was detectable in the expression of the four components of the MCU complex in FDB muscle of YS mice with reference to WT mice (Supplementary Figure S1).

### Caffeine-Induced Ca^2+^ Release From the SR: Reduced by Silencing MCU in YS, but Not in WT Fibers

**FIGURE 5:**
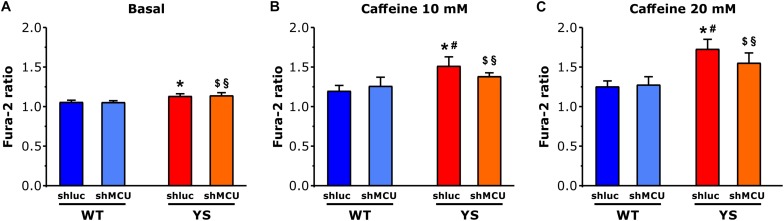
Cytosolic free Ca^2+^ concentrations determined as Fura-2 ratio following silencing of MCU. **(A–C)** Basal resting Ca^2+^ levels as evaluated by Fura 2 ratio during low frequency stimulation at 0.5 Hz is higher in YS fibers than in WT in the absence of caffeine **(A)** and in the presence of 10 mM caffeine **(B)** or 20 mM caffeine **(C)**. The increase of cytosolic calcium in YS fibers in response to 10 and 20 mM caffeine is reduced by silencing MCU. Four groups of fibers are compared: WT fibers transfected with shMCU (silencing), WT fibers transfected with shluc (control transfection), YS fibers transfected with shMCU (silencing), and YS fibers transfected with shluc (control transfection). Data are shown as means ± SEM; *p* < 0.05: ^∗^YS shluc vs. WT shluc and WT shMCU; ^$^YS shMCU vs. WT shluc; ^§^YS shMCU vs. WT shluc; and ^#^YS shluc vs. YS shMCU.

To test the hypothesis that mitochondria contribute to enhancement of Ca^2+^ release from SR via mutated RyR1 channel in YS muscle fibers, we silenced MCU to lower the Ca^2+^ entry in the mitochondrial matrix. Silencing of MCU was achieved by plasmid transfection with shMCU in FDB muscles of both YS and WT mice, while YS and WT muscles transfected with shluc were used as control. As can be seen in Figure 5, in WT fibers the amplitude of the cytosolic Ca^2+^ transient following exposure to 10 or 20 mM caffeine concentrations was unaffected by the silencing of MCU (as previously shown by Mammucari et al., 2015). In contrast, in YS fibers the knockdown of MCU partially, but significantly, reduced the cytosolic Ca^2+^ transient which follows the caffeine-induced Ca^2+^ release, an indication that blocking Ca^2+^ entry into mitochondria can reduce caffeine-induced Ca^2+^ release from SR in YS fibers.

### ROS Production in Mitochondrial Matrix: Enhanced in YS Fibers Compared to WT

**FIGURE 6:**
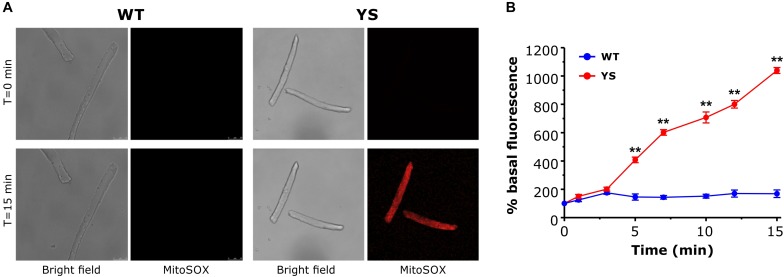
Mitochondrial ROS production in the presence of 20 mM caffeine as determined by MitoSox Red. **(A)** Representative bright field and Mitosox Red fluorescence images of single FDB fibers exposed to 20 mM caffeine at *T* = 0 and *T* = 15 min. **(B)** Average variations of MitoSox Red signal during exposure to 20 mM caffeine. Caffeine was added in one shot at *T* = 0. Data are shown as means ± SEM. ^∗∗^*p* < 0.01. *n* = 5 WT and 5 YS fibers.

Excessive production of oxidative species in YS muscles has been proposed as a key molecular event in the feed-forward cycle leading to contracture and rhabdomyolysis of skeletal fibers during MH crisis (Durham et al., 2008). We investigated whether in YS fibers the increased mitochondrial Ca^2+^ uptake during exposure of single FDB fibers to 20 mM caffeine (Figure 3) is accompanied by a greater generation of ROS. Experiments were carried out using MitoSox, a specific probe designed to evaluate superoxide ion generated in the mitochondrial matrix (Robinson et al., 2008; Pearson et al., 2014). We observed that a robust generation of ROS in mitochondria of FDB fibers of YS, but not of WT mice, followed caffeine administration (Figure 6) and, thus, accompanied the mitochondrial Ca^2+^ wave shown in Figure 3E. The accumulation of ROS continued to increase for the full duration of the record (up to 15 min).

### ROS Accumulation in the Mitochondrial Matrix: Reduced by MCU Silencing in YS, but Not in WT Fibers

**FIGURE 7:**
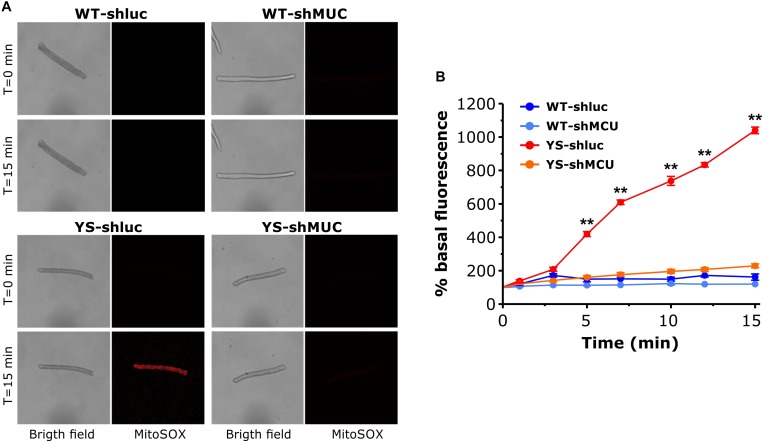
Mitochondrial ROS production as determined by MitoSox Red in the presence of 20 mM caffeine following silencing of MCU. **(A)** Representative bright field and Mitosox Red fluorescence images of single FDB fibers exposed to 20 mM caffeine at *T* = 0 and *T* = 15 min. For each group the same fiber is shown in bright field, ZsGreen fluorescence, used as marker of plasmid expression, and MitoSox Red fluorescence, used as probe for mitochondrial ROS. **(B)** Average variations of MitoSox Red signal during exposure to 20 mM caffeine. Caffeine was added in one shot at *T* = 0. Data are shown as means ± SEM. ^∗∗^*p* < 0.01. *n* = 5 fibers for each group.

We next asked whether the impact of MCU silencing on caffeine-induced Ca^2+^ release shown in Figure 5 could be mediated by changes in ROS generation in the mitochondria. We determined ROS in the mitochondrial matrix with Mitosox in muscle fibers transfected with plasmids carrying shMCU or shluc. The results are reported in Figure 7. As can be seen in Figure 7, knock down of MCU caused a marked reduction of ROS accumulation in the mitochondrial matrix. In YS fibers exposed to 20 mM caffeine, ROS levels (determined by MitoSox) were still high after transfection with shluc, but were reduced to the values of WT fibers after transfection with shMCU.

### Release of Ca^2+^ Induced by Increasing Caffeine Concentrations: Reduced by MitoTEMPO in YS, but Not in WT Fibers

**FIGURE 8:**
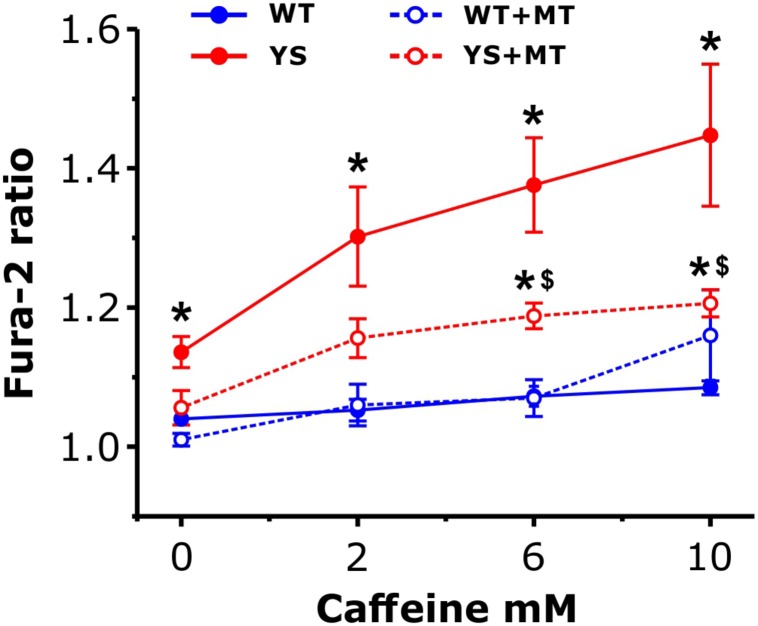
Cytosolic free Ca^2+^ concentrations in response to caffeine following pre-treatment with MitoTEMPO. Cytosolic Ca^2+^ levels, determined as Fura-2 ratio, during exposure to increasing caffeine concentrations in control YS and WT fibers and in YS and WT fibers pre-treated with MitoTEMPO (MT) for 24 h. Data are shown as means ± SEM; ^∗^*p* < 0.05, YS pre-treated and YS not pre-treated vs. WT; ^$^*p* < 0.05, YS pre-treated vs. YS not pre-treated. *n* = 5 fibers in each group.

Next, we sought to further confirm the contribution of the mitochondrial ROS generation to the mechanism leading to Ca^2+^ release in YS muscle by selectively removing the mitochondrial component of ROS production. To this end, we tested the effects of MitoTEMPO, a specific scavenger of mitochondrial superoxide, on a dose–response curve to Caffeine (from 0 to 10 mM). The levels of cytosolic [Ca^2+^] were taken as a proxi for the Ca^2+^ release from SR via RyR1. The results reported in Figure 8 showed that: (i) in untreated YS fibers the increase in cytosolic [Ca^2+^] was significantly greater than in WT fibers starting from 2 mM of caffeine and (ii) pretreatment for 24 h with MitoTEMPO was sufficient to keep cytosolic [Ca^2+^] lower in treated YS fibers than in untreated YS fibers even in the presence of 10 mM of caffeine. Pre-treatment with MitoTEMPO for 1 h was not sufficient to avoid the large Ca^2+^ accumulation in the cytosol (Supplementary Figure S2).

## Discussion

### Main Findings

Mice carrying a YS mutation of the amino acid 524 (522 in humans) of the Ca^2+^ channel RyR1 have provided for more than a decade an accepted model to study a group of human diseases: MH, environmental-exertional heat stroke (EHS), and central core disease (CCD) (Chelu et al., 2006; Durham et al., 2008; Boncompagni et al., 2009a; Michelucci et al., 2017a). MH and EHS are characterized by acute events of Ca^2+^ release from SR via the mutated channel, which is supported by the nitrosylation of RyR1 due to ROS/RNS accumulation in muscle fibers (Durham et al., 2008; Lanner et al., 2012). The link between the Ca^2+^ release through the mutated channel and the increased ROS/RNS generation has not been fully elucidated. While the role of NOX has been well demonstrated (Lanner et al., 2012), the contribution of mitochondria to ROS/RNS production is still unclear. Here we show that in YS muscle fibers (i) the increased Ca^2+^ accumulation in the mitochondrial matrix is detectable both at rest and after contractions (Figure 3); (ii) mitochondria are important sources of ROS during caffeine-induced contractures (Figure 6); (iii) ROS generation is dependent on Ca^2+^ entry in the mitochondria (Figure 7); and (iv) ROS generation in mitochondria is relevant to the increased Ca^2+^ release during the caffeine contracture (Figure 8).

### Elevated Cytosolic Ca^2+^ and SR Ca^2+^ Release in YS Fibers

In the present study, we measured free Ca^2+^ concentrations at rest and during contractile activity in three intracellular compartments: cytosol, SR lumen, and mitochondrial matrix (see Marcucci et al., 2018). In partial agreement with previous observations (Durham et al., 2008), we found that the resting basal free cytosolic Ca^2+^ concentration was higher in YS than in WT fibers. While the peak of cytosolic Ca^2+^ concentration during the transient triggered by a single action potential was not different between YS and WT, values significantly higher in YS were detected at the end of 2 s repetitive electrical 60 Hz stimulation and after exposure to 20 mM caffeine.

### SR Depletion in YS Fibers

We measured intraluminal free Ca^2+^ concentration of the SR and found that initial and final concentrations were lower in YS than in WT fibers. This finding is in agreement with the observations of Rios and colleagues (Manno et al., 2013), although the experimental protocol was different. Instead of voltage clamp, in the present study a train of electrical pulses was applied, with 2 s duration and high frequency (60 Hz) which is sufficient to reliably detect a decrease in intraluminal concentration (Canato et al., 2010). In this study, we analyzed also the impact of the Ca^2+^ release induced by caffeine and found again a depletion of SR much more pronounced in YS compared to WT. Depletion of the SR is a key signal for the activation of store operated Ca^2+^ entry (SOCE) (Kurebayashi and Ogawa, 2001; Michelucci et al., 2018), a mechanism that allows Ca^2+^ entry into the cytosol from the extracellular space. SOCE, if activated, could contribute to the elevated cytosolic resting Ca^2+^ levels. Indeed, Ca^2+^ entry from extracellular space has been proposed to contribute to altered Ca^2+^ homeostasis in human muscle fibers of MH susceptible patients (Duke et al., 2010) and in murine models of MH (Estève et al., 2010; Eltit et al., 2013; Yarotskyy et al., 2013). As suggested by Rios and coworkers (Manno et al., 2013), a frequent or even continuous activation of SOCE could explain the increased resting basal [Ca^2+^] in the cytosol.

### Elevated Mitochondrial Ca^2+^ Levels and ROS Generation in YS Fibers

We measured the free Ca^2+^ concentration in the mitochondrial matrix of YS fibers and found that it was significantly higher in YS fibers than in WT at rest, during electrical stimulation, and after caffeine administration. Due to their specific location close to the Ca^2+^ release units (Rudolf et al., 2004; Boncompagni et al., 2009b; Raffaello et al., 2016), mitochondria are very sensitive to Ca^2+^ leakage from SR as well as to the release under electrical or chemical (caffeine) stimulation. Mitochondria are also not far from the specific structures involved in SOCE (Boncompagni et al., 2017) and, as discussed above, calcium entry via SOCE is likely activated in YS muscle fibers. In contrast, an increased availability of mitochondrial Ca^2+^ transport mechanism seems unlikely as suggested by the analysis of the expression of MCU components (Figure 4).

The free Ca^2+^ concentration in the mitochondrial matrix is a signal for metabolic regulation (see for a review Rizzuto et al., 2012) based on activation of pyruvate, isocitrate, 2-oxoglutarate dehydrogenases (Denton, 2009), and oxidative phosphorylation cascade (Glancy et al., 2013). Thus, higher levels of mitochondrial Ca^2+^ could contribute to the hypermetabolic condition, which is typical of YS mice exposed to anesthetics, heat, or physical exertion (Chelu et al., 2006; Michelucci et al., 2017a). In addition, higher levels of Ca^2+^ in the mitochondrial matrix could also be responsible of the observed increased accumulation of ROS in mitochondria of YS muscle fibers (Figures 6, 7). In full agreement with previous findings on electrical stimulation (Michaelson et al., 2010; Pearson et al., 2014), our data show that during and after the wave of Ca^2+^ release associated with caffeine administration (Figure 1E), the generation of ROS in the mitochondria is minimal in WT fibers. In contrast, in YS fibers a large and long lasting ROS accumulation, detected by Mitosox, accompanies the calcium release induced by caffeine (Figure 6). This finding is consistent with the observation of a greater temperature-dependent generation of mitochondrial superoxide flashes (mSOF) in YS fibers (Wei et al., 2011). Both the temperature- and caffeine-dependent increase in ROS production in YS fibers could be a direct consequence of higher concentration of free Ca^2+^ in the mitochondrial matrix. Additionally, the mitochondrial cristae remodeling observed by Boncompagni et al. (2009a) could imply supercomplexes destabilization that in turn could facilitate ROS production (Cogliati et al., 2013). It is worth to note that the mitochondrial ROS accumulation seems to require a long time period with high Ca^2+^ concentration and, thus, it shows up only in the presence of sufficient (above threshold) caffeine concentration. The gradual and long lasting increase of Mitosox fluorescence induced by caffeine in YS fibers is also consistent with previous observations on mitochondrial ROS (Pearson et al., 2014).

We hypothesize that the generation of ROS might lead to formation of RNS which are responsible for the nitrosative modifications and further activation of calcium release in RyR1, thus generating a feed forward cycle (Durham et al., 2008; Supplementary Figure S3). In support of this view, here we showed that MCU silencing, which dramatically lowers Ca^2+^ entry in the mitochondrial matrix (Mammucari et al., 2015), was followed by a marked reduction of mitochondrial ROS accumulation in YS fibers to levels similar to WT (Figure 7). This was accompanied by a significant reduction in cytosolic Ca^2+^ levels and normalization of sensitivity of YS fibers to caffeine-induced Ca^2+^ release (Figure 5). Furthermore, the MitoTEMPO-dependent reduction of ROS in the mitochondria of YS fibers effectively lowered both caffeine-induced Ca^2+^ release and cytosolic Ca^2+^ levels (Figure 8) strongly suggesting that the mitochondrial contribution is relevant to enhance Ca^2+^ release in YS muscle fibers in response to caffeine, likely via oxidative/nitrosative modification of RyR1.

## Conclusive Remarks

The results reported in this study point to the increased mitochondrial Ca^2+^ uptake as the link between SR leak and generation of ROS/RNS in the mitochondrial matrix. These findings suggest that inhibitors of Ca^2+^ entry into mitochondria could be tested as alternative pharmacological approaches to verify if they could be as effective in blocking MH crises as antioxidant treatments (e.g., NAC and Trolox; Durham et al., 2008; Michelucci et al., 2015) and compounds controlling Ca^2+^ release dantrolene, azumulene (Michelucci et al., 2017b), and AICAR (Lanner et al., 2012).

## Author Contributions

CR, MC, and PC designed the experiments. MC, PC, LC, LL, and DV performed the experiments. MC, PC, LM, and CR analyzed the data. LM, AR, AM, CR, and FP discussed the data. CR and FP wrote the manuscript.

## Conflict of Interest Statement

The authors declare that the research was conducted in the absence of any commercial or financial relationships that could be construed as a potential conflict of interest.

## Supplementary Material

FIGURE S1 Quantitative determination of the expression levels of four components of MCU complex as obtained with Q-PCR. Levels in WT muscles are taken as reference.

FIGURE S2 Cytosolic calcium concentration determined as fluorescence ratio of Fura-2 in WT and YS muscle fibers exposed to caffeine with or without pre-treatment with MitoTempo (MT) for 1 h before the actual experiment. The difference between YS and WT is highly significant, while no difference is detectable between treated and untreated muscle fibers.

FIGURE S3 Model for ROS generation in YS muscle fibers during contractile activity. We propose that the greater response of RyR1^*Y524S*^ to caffeine implies not only a greater increase in cytosolic calcium concentration but also a greater calcium uptake by mitochondria. The latter is followed by a significant generation of ROS in the mitochondria, which contributes to RyR nitrosylation together with ROS generation in the cytosol. RyR nitrosylation, in turn, induces a further increase in RyR1 permeability, thus creating a positive feed back loop. Two interventions, reduction of calcium uptake *via* MCU silencing and control of ROS accumulation with mitotempo, prove to be sufficient to interrupt the positive feed back loop.

## Abbreviations

EC-coupling: excitation–contraction coupling
FDB: flexor digitorum brevis
MH: malignant hyperthermia
ROS and RNS: reactive oxygen species and reactive nitrogen species
RyR1: ryanodine receptor type-1
SR: sarcoplasmic reticulum
WT: wild type.
