# Supplementary figures and images for: Excessive Accumulation of Ca2 + in Mitochondria of Y522S-RYR1 Knock-in Mice: A Link Between Leak From the Sarcoplasmic Reticulum and Altered Redox State
Source: Front Physiol. 2019 Sep 13;10:1142. doi: 10.3389/fphys.2019.01142 (PMC6755340; doi:10.3389/fphys.2019.01142)

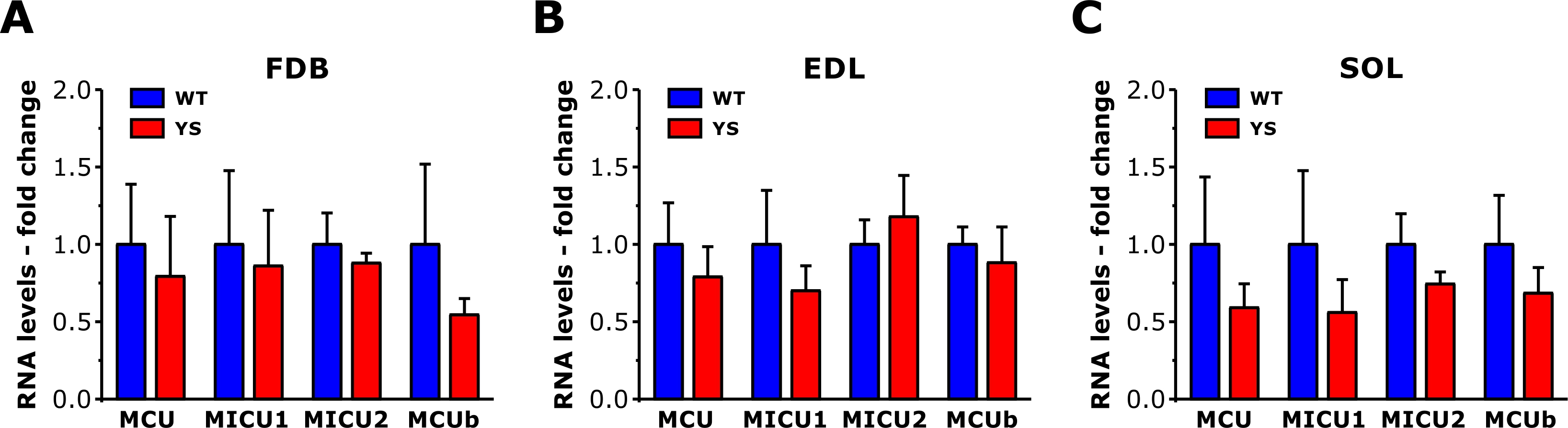

Supplement: FIGURE S1 — Quantitative determination of the expression levels of four components of MCU complex as obtained with Q-PCR. Levels in WT muscles are taken as reference. [file Image_1.JPEG]

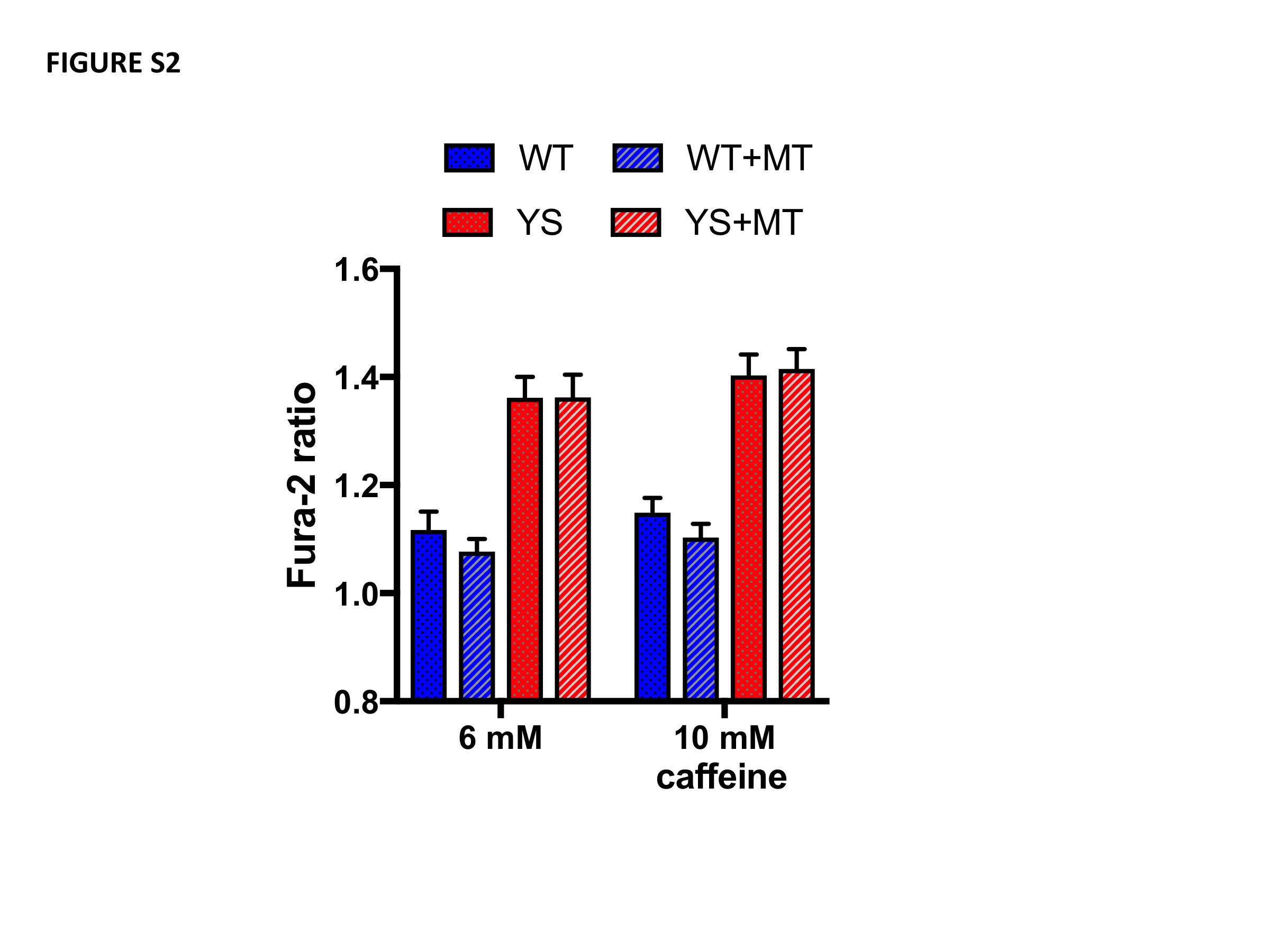

Supplement: FIGURE S2 — Cytosolic calcium concentration determined as fluorescence ratio of Fura-2 in WT and YS muscle fibers exposed to caffeine with or without pre-treatment with MitoTempo (MT) for 1 h before the actual experiment. The difference between YS and WT is highly significant, while no difference is detectable between treated and untreated muscle fibers. [file Image_2.JPEG]

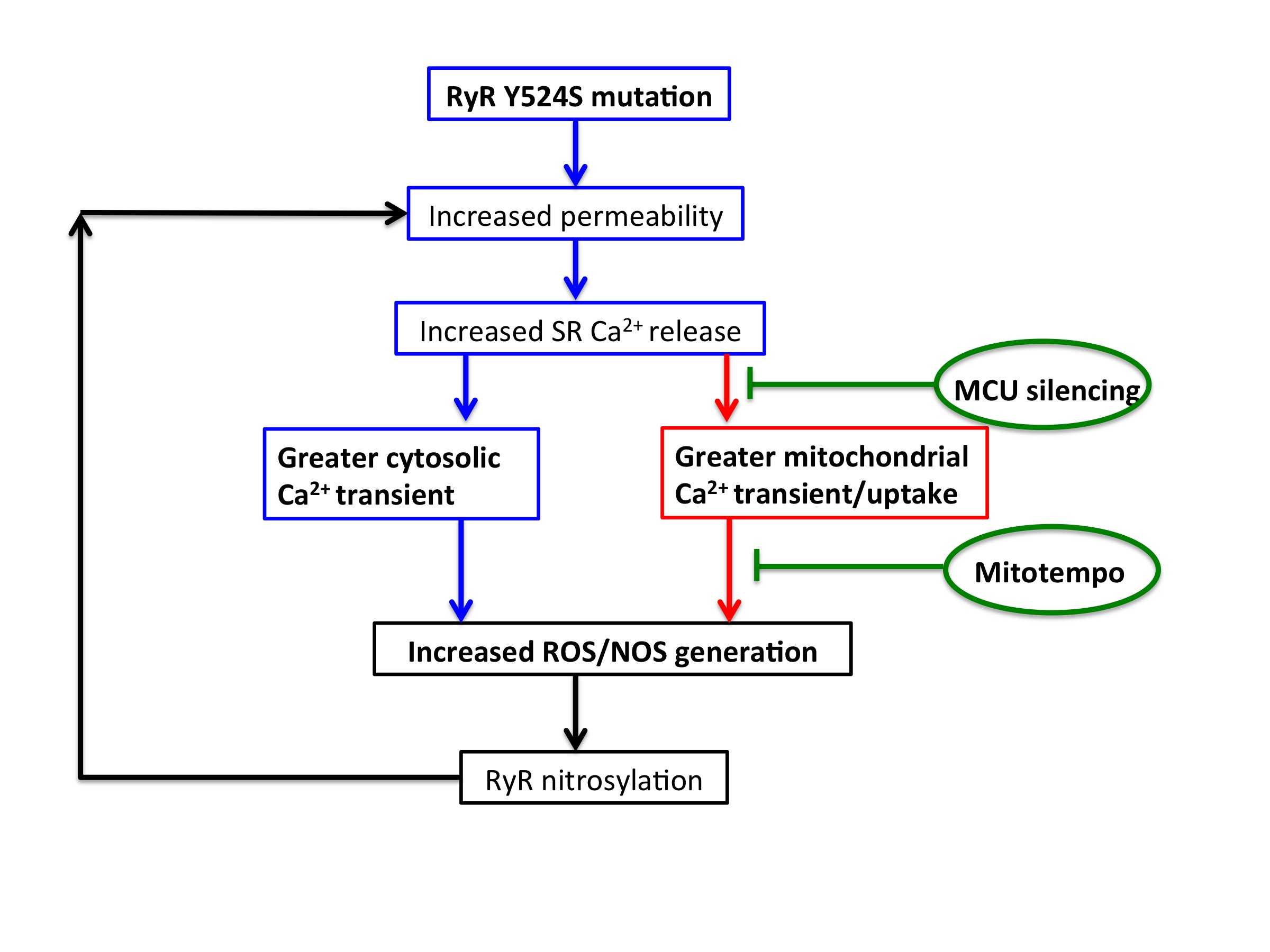

Supplement: FIGURE S3 — Model for ROS generation in YS muscle fibers during contractile activity. We propose that the greater response of RyR1Y524S to caffeine implies not only a greater increase in cytosolic calcium concentration but also a greater calcium uptake by mitochondria. The latter is followed by a significant generation of ROS in the mitochondria, which contributes to RyR nitrosylation together with ROS generation in the cytosol. RyR nitrosylation, in turn, induces a further increase in RyR1 permeability, thus creating a positive feed back loop. Two interventions, reduction of calcium uptake via MCU silencing and control of ROS accumulation with mitotempo, prove to be sufficient to interrupt the positive feed back loop. [file Image_3.JPEG]
